# A Case of Metastatic Hereditary Leiomyomatosis and Renal Cell Cancer Syndrome-Associated Renal Cell Carcinoma Treated with a Sequence of Axitinib and Nivolumab Following Cytoreductive Nephrectomy

**DOI:** 10.15586/jkcvhl.2020.148

**Published:** 2020-07-20

**Authors:** Ichiro Yonese, Masaya Ito, Kosuke Takemura, Takao Kamai, Fumitaka Koga

**Affiliations:** 1 Department of Urology, Tokyo Metropolitan Cancer and Infectious Diseases Center, Tokyo, Japan; 2 Department of Urology, Dokkyo Medical University School of Medicine, Tochigi, Japan

## Introduction

Hereditary leiomyomatosis and renal cell cancer syndrome (HLRCC) is an autosomal dominant condition, characterized by the development of cutaneous leiomyomas, multiple uterine leiomyomas, and an aggressive form of type 2 papillary renal cell carcinoma (RCC) (1–3). HLRCC is caused by germline mutation of the *fumarate hydratase (FH)* gene, and about 15% of individuals with *FH* mutations develop HLRCC-associated RCC (4). This type of RCC is aggressive with a median overall survival of 15.8 months for stage III or stage IV disease (3). Because of its rarity, no standard treatment has been established for advanced or metastatic disease. We report a case of advanced HLRCC-associated RCC, in which clinical features mimicked advanced renal pelvic cancer, and long-term survival was achieved with cytoreductive nephrectomy (CN), followed by a sequence of axitinib and nivolumab. Although there is no prior clinical evidence, the molecular and biological characteristics of the disease support therapeutic roles for these treatments in the management of metastatic HLRCC-associated RCC.

## Case Report

**Figure 1:**
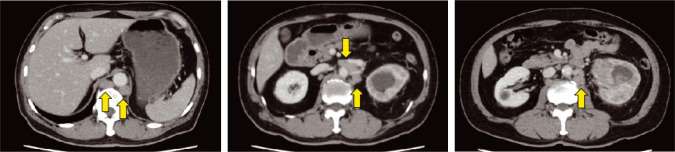
Computed tomography at the first visit. A moderately enhanced mass occupies the left renal pelvis, accompanying hydronephrosis and multiple para-aortic lymphadenopathy (arrows).

A 65-year-old Japanese male diagnosed with left renal pelvic cancer was referred to us. He had visited another hospital due to gross hematuria. Incidentally, his daughter had undergone nephrectomy for kidney cancer at the age of 36 years. Physical examinations, including the skin, revealed no abnormal findings. Laboratory findings showed hematuria, elevated serum creatinine (1.47 mg/dL), and C-reactive protein (12.1 mg/L). Urine cytology showed atypical urothelial cells. Computed tomography (CT) scans identified a moderately enhanced mass occupying the left renal pelvis, in addition to hydronephrosis, multiple para-aortic lymphadenopathy, and left adrenal swelling (Figure 1).

Differential diagnosis included urothelial carcinoma of the renal pelvis, collecting duct carcinoma, and other rare types of RCC, which were less likely. Considering that locally advanced disease of either urothelial carcinoma of the renal pelvis or collecting duct carcinoma was probable, the patient was started on systemic chemotherapy with gemcitabine and cisplatin. However, his disease progressed slowly after six courses (7 months) of chemotherapy.

**Figure 2:**
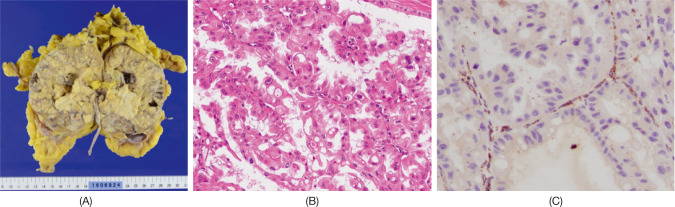
Pathological findings of nephrectomy specimen. Macroscopic findings (A), microscopic findings (B), hematoxylin and eosin stain, ×200), and immunohistochemistry of fumarate hydratase (C, ×200).

**Figure 3:**
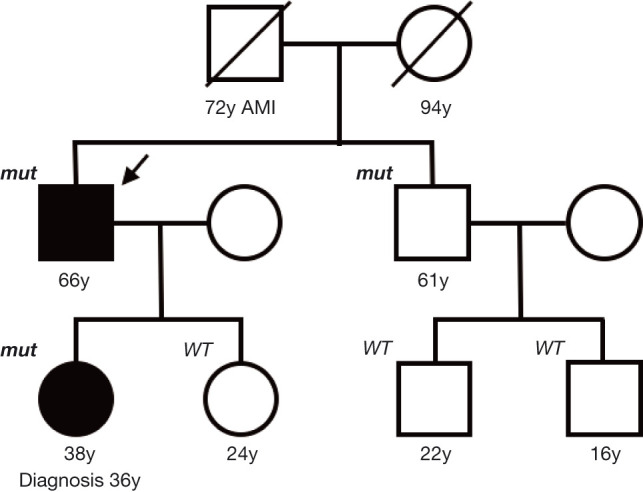
Family pedigree. Ages shown are those at which subjects took genetic testing. An arrow indicates the present case. AMI, acute myocardial infarction; *mut*, germline mutation of the *fumarate hydratase* gene; WT, wild type.

Owing to the poor response of the tumor to chemotherapy with gemcitabine and cisplatin, we considered the probability of rare types of RCC and proposed nephrectomy for pathological diagnosis along with mass reduction. The patient underwent CN with sampling of renal hilar lymphadenopathy with an uneventful postoperative course. Grossly, the tumor comprised multiple nodules spreading over the renal parenchyma and a solid pelvic mass (Figure 2A). Microscopically, the tumor consisted of carcinoma cells with abundant eosinophilic cytoplasm and large nuclei arranged in a papillary architecture, which was consistent with type 2 papillary RCC (Figure 2B). We suspected HLRCC-associated RCC because of the patient’s family history. Immunohistochemistry revealed no expression of *FH* in carcinoma cells

(Figure 2C). Germline genetic testing revealed heterozygous *FH* deletion in exon 2 (c.247_267del), confirming the diagnosis of HLRCC-associated RCC. Finally, a pathological diagnosis of HLRCC-associated RCC, pT3apN2 (stage IV), was made. The same *FH* mutation was also found in his daughter with a previous history of kidney cancer and his brother among five relatives examined (Figure 3).

**Figure 4:**
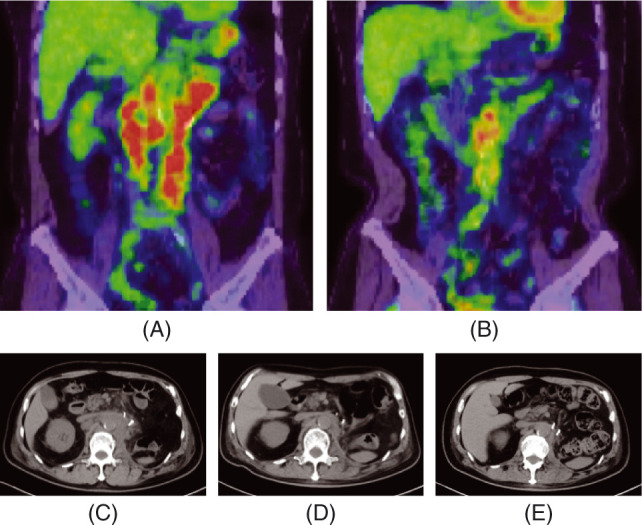
FDG-PET before axitinib (A) and 8 months after nivolumab following 7-months of axitinib (B). Metabolic tumor volume (MTV) was defined as the tumor volume greater than 40% of standardized uptake value (SUV) max. Total lesion glycolysis (TLG) was calculated as MTV×SUVmean. MTV and TLG were calculated by using LIFEx package (www.lifexsoft.org). MTV/TLG are 215.3 cm^3^/1042.5 g (A) and 15.8 cm^3^/112.5 g (B). CT before axitinib (C), 6 months after axitinib (D), and 7 months after nivolumab (E).

To assess the metabolic activity of HLRCC-associated RCC, fluorodeoxyglucose–positron emission tomography (FDG-PET) was performed. It showed strong accumulation in para-aortic lymph nodes, the left adrenal gland, and focally in the 12th thoracic to the third lumbar vertebrae. Metabolic tumor volume (MTV) and total lesion glycolysis (TLG) were 215.3 cm^3^ and 1042.5 g, respectively (Figure 4A). Axitinib was initiated following radiotherapy at 30 Gy in 10 fractions in combination with zoledronic acid to bone metastases, which achieved a partial response (Figure 4C, 4D). Nivolumab was started 7 months after starting axitinib. The patient had been in sustained partial response on nivolumab, with significant reductions in MTV and TLG of 15.8 cm^3^ and 112.5 g, respectively, at 8 months after the start of nivolumab (Figure 4B, 4E). However, bilateral adrenal metastases progressed 18 months after the start of the systemic sequential therapy. He re-started axitinib (progressive disease 10 months later), followed by everolimus (stable disease but discontinued due to hepatic and renal toxicities 2 months later) and re-challenge of nivolumab. He is alive with disease on nivolumab beyond slowly progressive disease 48 months after CN (58 months after the diagnosis of advanced kidney cancer).

## Discussion

It is challenging to differentiate infiltrative nonclear cell renal cell carcinoma (nccRCC) accompanied by hydronephrosis from invasive renal pelvic cancer and collecting duct carcinoma. In the present case, systemic chemotherapy was initially given based on the clinical diagnosis of advanced urothelial carcinoma or collecting duct carcinoma, which eventually delayed treatment for advanced HLRCC-associated RCC. Biopsy should be considered with a possible diagnosis of hereditary RCC in mind in the case of a positive family history of juvenile kidney cancer, although other clinical features indicate renal pelvic cancer or collecting duct carcinoma.

Genetic testing and counseling should be offered to at-risk family members. Given the aggressive nature of HLRCC-related RCC, individuals harboring a pathogenic *FH* mutation should be placed on surveillance, including annual renal magnetic resonance imaging (3). Our patient’s healthy brother with a germline *FH* mutation is also on surveillance.

Upfront, CN is currently not recommended for all of metastatic RCC patients treated with tyrosine kinase inhibitors (TKIs), based on the results of two randomized trials for metastatic clear cell RCC (ccRCC) (5, 6). As for metastatic papillary RCC, in which TKIs were less effective than in ccRCC, a prognostic benefit of CN was reported in spite of a retrospective study using the International Metastatic RCC Database Consortium (IMDC) database (7). No data are available on the prognostic role of CN for metastatic HLRCC-associated RCC.

Biologically, HLRCC-associated RCC is characterized by the accumulation of fumarate, which induces subsequent stabilization of hypoxia-inducible factors (HIFs) (8) and nuclear factor E2-related factor 2 (NRF2) accumulation (9), leading to a Warburg-like metabolic shift to glycolysis-dependent metabolism (2, 10). In fact, HLRCC-associated RCC shows remarkably high glucose uptake on FDG-PET (11),. Of note, pretreatment MTV and TLG have been reported as prognostic biomarkers in metastatic RCC patients treated with TKIs; increased MTV and TLG are reportedly associated with worse prognosis (12). In our case, CN was conducted mainly for diagnostic purpose. Given that TKIs are potentially effective for metastatic HLRCC-associated RCC, as discussed later, CN or other debulking surgery may have a prognostic role by reducing MTV and TLG prior to TKI therapy.

No standard of care has been established for metastatic HLRCC-associated RCC. With the concept of inhibiting HIF-dependent angiogenesis and glucose uptake by dual blockade of vascular endothelial growth factor receptor/epidermal growth factor receptor, bevacizumab and erlotinib are being tested in a phase II clinical trial (NCT01130519). In the first 20 patients with HLRCC-associated RCC, an overall response rate of 65% was reported (13). A randomized phase II study comparing a c-MET inhibitor cabozantinib with sunitinib for metastatic papillary RCC is also ongoing (NCT02761057). Axitinib was administered in two cases of HLRCC-associated RCC as an alternative systemic therapy and was found to be effective in one case (14, 15); a patient with regional lymph node metastasis receiving axitinib in a neoadjuvant setting showed major pathological response and remained disease-free for over 33 months after radical nephrectomy and regional lymph node dissection (14). In the present case, axitinib following CN was also effective; treatment for a period of 7 months profoundly reduced the tumor volume.

Although immune checkpoint inhibitors (ICIs) are currently the standard of care for metastatic RCC, there is limited evidence on their efficacy for nccRCC. The overall response rate of ICIs for nccRCC was about 20% (16, 17). In three young cases of papillary RCC, nivolumab achieved a durable response (18–20). However, no data are available on the activity of ICIs for HLRCC-associated RCC. In general, higher tumor mutation burden (TMB) and a resultant increase in tumor neoantigens are associated with better outcomes with ICIs (21). In spite of the moderate TMB measured by whole exon sequencing, frequent frameshift insertions and deletions produce more neoantigens than expected according to the TMB in RCC, irrespective of the histological type (22). In HLRCC-associated RCC, accumulating fumarate impairs homologous recombination DNA repair (23), increasing insertions and deletions, and consequently increasing the number of neoantigens (22). In addition, the majority of HLRCC-associated RCC cases are categorized as CpG island methylation phenotype (CIMP) based on molecular profiling (24), which is characterized by increased immune cell infiltrate gene expression signature similar to ccRCC, thereby eliciting a favorable response to ICIs (24). These molecular characteristics suggest the efficacy of ICIs for HLRCC-associated RCC.

In our case, nivolumab sustained stable disease for 11 months after the tumor volume had been significantly reduced by axitinib. Lower MTV on FDG-PET was reported to be associated with more favorable outcomes of ICIs in advanced melanoma (25) and NSCLC (26). It remains to be elucidated whether that is the case or not in metastatic RCC.

In the present case, long-term survival (48 months from CN and 58 months from the diagnosis of advanced kidney cancer) was achieved through multidisciplinary sequential treatment consisting of CN, radiation, axitinib, and nivolumab. Given that all nine patients with stage III or stage IV HLRCC-associated RCC in a previous report died within 3 years (3) and the median survival was about 1 year for CIMP-RCC patients (24), the multidisciplinary sequential treatment seems to have prolonged survival in our case. Further investigations are needed to evaluate the efficacy of such a therapeutic strategy for this rare and aggressive disease.

## Conclusion

We experienced a case of metastatic HLRCC-associated RCC, in which clinical features mimicked advanced renal pelvic cancer. Biopsy should be considered with a possible diagnosis of hereditary RCC in mind, particularly in the case of a positive family history of juvenile kidney cancer. In this case, long-term survival was achieved with multimodal sequential treatment consisting of CN, radiation to bone metastases, axitinib, and nivolumab. In spite of no prior clinical evidence, the molecular and biological characteristics of the disease support therapeutic roles for these treatments in the management of metastatic HLRCC-associated RCC.
